# New enumeration algorithm for protein structure comparison and classification

**DOI:** 10.1186/1471-2164-14-S2-S1

**Published:** 2013-02-15

**Authors:** Cody Ashby, Daniel Johnson, Karl Walker, Iyad A Kanj, Ge Xia, Xiuzhen Huang

**Affiliations:** 1 Molecular Bioscience Graduate Program, Arkansas State University, Arkansas, USA; 2 Bioinformatics Graduate Program, University of Arkansas at Little Rock, Arkansas, USA; 3 School of Computing, DePaul University, Illinois, USA; 4 Department of Computer Science, Lafayette College, Pennsylvania, USA; 5 Department of Computer Science, Arkansas State University, Arkansas, USA

## Abstract

**Background:**

Protein structure comparison and classification is an effective method for exploring protein structure-function relations. This problem is computationally challenging. Many different computational approaches for protein structure comparison apply the secondary structure elements (SSEs) representation of protein structures.

**Results:**

We study the complexity of the protein structure comparison problem based on a mixed-graph model with respect to different computational frameworks. We develop an effective approach for protein structure comparison based on a novel independent set enumeration algorithm. Our approach (named: ePC, **e**fficient **e**numeration-based **P**rotein structure **C**omparison) is tested for general purpose protein structure comparison as well as for specific protein examples. Compared with other graph-based approaches for protein structure comparison, the theoretical running-time *O*(1.47*^rn^n*^2^) of our approach ePC is significantly better, where *n *is the smaller number of SSEs of the two proteins, *r *is a parameter of small value.

**Conclusion:**

Through the enumeration algorithm, our approach can identify different substructures from a list of high-scoring solutions of biological interest. Our approach is flexible to conduct protein structure comparison with the SSEs in sequential and non-sequential order as well. Supplementary data of additional testing and the source of ePC will be available at http://bioinformatics.astate.edu/.

## Background

Protein structure comparison is an effective method for exploring protein structure-function relations and for studying evolutionary relations of different species. It can also be applied to identify the active sites of carrier proteins, the binding sites of antibodies, the inhibition sites of enzymes, and the common structural motifs of proteins, which has significant applications in biological and biomedical research.

The computational methods for protein structure comparison usually represent a protein structure by atomic coordinates in the Euclidean space, as a distance matrix [1] whose entries represent the distances between two residues of the protein, or as a contact map [2], where a binary matrix is used to represent the distances between the residue pairs. A structure graph representation of a protein tertiary structure was first defined in [3] for protein structure prediction. In this current work, we adopt the structure graph representation in [3]. We develop a very efficient graph-based approach for protein structure comparison. Our approach transforms the comparison problem to an independent set problem in an auxiliary graph, and then applies a novel enumeration algorithm to identify the best out of a set of good comparison candidates.

We first show the problem of comparing a query structure to another structure is intractable with respect to several computational frameworks. For example, we show that the problem is NP-hard (even for very restricted instances), cannot be approximated to a ratio $n^{\frac{1}{2}-\varepsilon }$, for any *ε *> 0, unless P=NP, and is *W *[1]- complete with respect to the framework of parameterized complexity. We also show that a useful case of the problem is solvable in polynomial time by reducing it to the 2-CNF-SATISFIABILITY problem.

Whereas the above results are negative hinting at the challenging nature of the problem, the graph-based approach we use allows us to model the problem as a maximum independent set problem, for which a repertoire of effective exact algorithms exist in the literature. We use an algorithm developed by (some of) the authors [4] to enumerate the top-*K *maximum independent sets in a graph in time *O*(1.47*^n^n*^2^), where *n *is the number of vertices in the graph (Note that the algorithm in [4] enumerates the top-*K *minimum vertex covers in a graph, but obviously can be used to enumerate the top-*K *maximum independent sets in a graph using the standard reduction between vertex cover and independent set); this enumeration algorithm allows us to sift through the top SSE alignments for the protein structure comparison problem, looking for the best amongst them in terms of accuracy. Compared with other graph-base approaches, the theoretical running-time *O*(1.47*^rn^n*^2^) of our approach ePC is the current best, where n is the smaller number of SSEs of the two proteins, *r *is an introduced parameter of small values.

Many different approaches for protein structure comparison apply the secondary structure elements (SSEs) representation and database searching, such as deconSTRUCT [5], SSM [6], GANGSTA [7], MASS [8,9], VAST [10], TOPS [11] and approaches in [12-19]. Our approach ePC utilizes the SSE-based representation of the protein structure, and takes into consideration the global 3D structural arrangements of the SSEs of the proteins. We compare our approach with two other SSE-based approaches: deconSTRUCT, an approach for general purpose protein structure comparison and database search, and SSM, a high-resolution structure comparison approach. Our approach has comparable performance as deconSTRUCT. With a more general and simplified representation and a unified graph enumeration algorithm, our approach could detect a substructure or motif structure in a set of large structures, more than one common substructure shared by a set of proteins. It is very flexible. Our approach could use a wide range of evaluation functions for protein structure comparison. It could be applied to handle sequential and non-sequential order of SSE alignment and be extended to handle challenging protein multiple structure alignment and protein subset alignment.

## Methods

**Figure 1:**
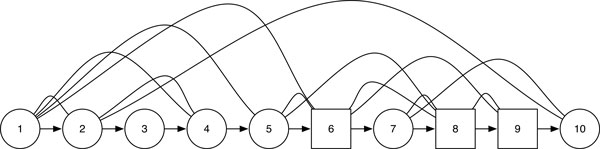
**Structure graph for 6ldh**. Alpha helix elements are represented by circles and beta strand elements are represented by squares.

A mixed graph for a protein structure is constructed from the PDB file as follows: each vertex represents a core/secondary structure element (i.e., an alpha helix element, or, a beta strand element), each undirected edge represents the interaction between two cores, and each directed edge (arc) represents the loop between two consecutive cores (from the N-terminal to the C-terminal). A mixed graph representation is used for protein structure prediction in [3]. The DSSP program [20,21] was used for the assignments of secondary structure elements for the protein entries from the Protein Data Bank (PDB). Refer to the protein structure and the corresponding mixed graph representation in Figures 1 for protein with ID: 6ldh. Alpha helix elements are represented by circles and beta strand elements are represented by squares. Therefore, a mixed graph can be represented as a triple *G *= (*V *(*G*), *A *(*G*), *E *(*G*)), where *V *(*G*) is the vertex-set of *G*, *E *(*G*) is the set of undirected edges of *G*, and *A *(*G*) is the set of directed edges of *G*, which induces a directed path spanning all vertices of *G*, thus defining a linear order among the vertices of *V *(*G*). The aforementioned mixed graph representation incorporates the SSE type, the sequential order of the SSEs, and the interactions of the SSEs. When comparing two protein structures, the problem could now be reduced to finding the common subgraph of the two mixed graph.

Goldman et al. [2] studied the protein comparison problem using the notion of *contact maps*. Contact maps are undirected graphs whose vertices are linearly ordered. Goldman et al. [2] formulated the protein comparison problem as a CONTACT MAP OVERLAP problem, in which we are given two contact maps and we need to identify a subset of vertices *S *in the first contact map, a subset of vertices $S^{'}$ in the second with $\left|S\right|=\;|S^{'}$, and an order preserving (w.r.t. linear ordering) bijection $f:S\to S^{'}$, such that the number of edges in *S *(i.e., between the vertices in *S*) that correspond (under *f*) to edges in $S^{'}$ is maximized. In [2], the authors proved that the CONTACT MAP OVERLAP problem is MAXSNP-complete even when both contact maps have maximum degree one.

Song et al. [3] studied the problem of mixed-graph comparison, when each vertex *v *in the first mixed-graph is associated with a subset of vertices *S_v _*in the second mixed-graph, and the bijection *f *is restricted to map *v *to a vertex in *S_v_*. Song et al. [3] proved that this problem is NP-complete, even when the size of each subset *S_v_*, referred to as the *map width *is at most 3. Our results in the following section refine and extend the results in [3] in several aspects. We first prove that the problem defined in [3] is intractable with respect to many computational frameworks. For example, we show that the problem: (1) is NP-hard (even for very restricted instances), (2) cannot be approximated to a ratio $n^{\frac{1}{2}-\varepsilon }$, for any *ε *> 0, unless P=NP, and (3) is W [1]-complete with respect to the framework of parameterized complexity. We also show that a useful case of the problem is solvable in polynomial time by reducing it to the 2-CNF-SATISFIABILITY problem.

### The graph embedding problem and complexity results

In this section, we study the complexity of the mixed graph embedding problem, which corresponds to the problem of identifying the query protein structure (e.g., a motif structure) as a substructure in a larger protein structure.

We define the GRAPH EMBEDDING problem as follows:

GRAPH EMBEDDING

Given two mixed graphs *G *= (*V *(*G*), *A*(*G*), *E*(*G*)) and *H *= (*V *(*H*), *A*(*H*), *E*(*H*)), where *H *is referred to as the *host graph*, such that each vertex *v *∈ *V *(*G*) has a list *L*(*v*) ⊆ *V *(*H*) of vertices in *H *that it can be mapped to, decide if there exists an injection *f*: *V *(*G*) → *V *(*H*) such that:

(i) *f *(*v*) ∈ *L*(*v*) for every *v *∈ *V *(*G*);

(ii) for any two vertices *v*, $\nu^{'}\in V\left(G\right)$, there is a directed path from *v *to $\nu^{'}$ in G if and only if there is a directed path from f(*v*) to $f\left(\nu^{'}\right)$ in *H*; and

(iii) for any two vertices *v*, $\nu^{'}\in V\left(G\right)$, if $\nu \nu^{'}\in E\left(G\right)$ then $f\left(\nu \right)f\left(\nu^{'}\right)\in E\left(H\right)$.

We shall call an injective embedding *f *satisfying properties (i)-(iii) above a *valid embedding*.

Informally speaking, the GRAPH EMBEDDING problem asks if we can embed *G *into *H *in such a way that the precedence order determined by the arcs of *G *is respected by this embedding, and the undirected edges of *G *are respected by this embedding.

We define the restriction of the GRAPH EMBEDDING problem, denoted *r*-GRAPH EMBEDDING, where *r *is positive integer, by restricting the cardinality of the set *L*(*v*) to be at most *r*, for every *v *∈ *V *(*G*); that is, in the restrictions of the problems, a vertex in *V *(*G*) can be mapped to at most *r *vertices in *H*.

If one cannot embed the whole graph *G *into *H*, it is natural to seek an embedding that embeds the maximum number of vertices in *G *into *H*, while respecting conditions (i)-(iii) above. Therefore, we define a version of GRAPH EMBEDDING, denoted GRAPH EMBEDDING_≥_, by introducing a nonnegative parameter *k*, and asking whether there exists a subset *S *⊆ *V *(*G*) with |*S*|≥ *k*, and an injection *f*: *S *→ *V *(*H*) such that:

(i) *f*(*v*) ∈ *L*(*v*) for every *v *∈ *S*;

(ii) for any two vertices *v*, $\nu^{'}\in S$, if there is a directed path from *v *to $\nu^{'}$ in *G *then there is a directed path from *f*(*v*) to $f\left(\nu^{'}\right)$ in *H*; and

(iii) for any two vertices *v*, $\nu^{'}\in S$, if $\nu \nu^{'}\in E\left(G\right)$ then $f\left(\nu \right)f\left(\nu^{'}\right)\in E\left(H\right)$.

The optimization/maximization version of the GRAPH EMBEDDING_≥ _problem, denoted MAXIMUM GRAPH EMBEDDING, asks for a set *S *of maximum cardinality that satisfies conditions (i)-(iii) above. Similarly, we can define the problems *r*-GRAPH EMBEDDING_≥ _and MAXIMUM r-GRAPH EMBEDDING.

It was shown in [3] that a more general problem than *r*-GRAPH EMBEDDING, in which the set of edges *A*(*G*) do not necessarily induce a path, is NP-complete for any *r *≥ 3. The same proof actually shows that the *r*-GRAPH EMBEDDING problem is NP-complete for any *r *≥ 3. We show next that the 2-GRAPH EMBEDDING is solvable in polynomial time.

**Theorem 0.1 ***The *2-GRAPH EMBEDDING *problem is solvable in polynomial time*.

PROOF. We reduce the problem to 2-CNF-SATISFIABILITY, which is solvable in polynomial time (for example, see [22]. Recall that in the 2-CNF-SATISFIABILITY problem we are given a Boolean formula in the *conjunctive normal form *(CNF) (i.e., the formula is the conjunction of clauses, and each clause is the disjunction of a literals, which are variables or negations of variables), in which each clause contains at most two literals, and we are asked to decide whether or not the formula is satisfiable. Let (*G*, *H*) be an instance of 2-GRAPH EMBEDDING satisfying |*L*(*v*)| ≤ 2, for every *v *∈ V (G). We show how to construct in polynomial time an instance *F *of 2-CNF-SATISFIABILITY such that *G *has a valid embedding into *H *if and only if *F *is satisfiable.

For every vertex *v *∈ *G*: if $L\left(\nu \right)=\left\{\nu^{'}\right\}$ we add a variable $x_{\nu \nu^{'}}$ and add the clause $\left\{x_{\nu \nu^{'}}\right\}$ to *F*; and if $L\left(\nu \right)=\left\{\nu^{'},\nu^{\prime \prime }\right\}$ we add the two variables $x_{\nu \nu^{'}}$, $x_{\nu \nu^{\prime \prime }}$and the two clauses $\left\{x_{\nu \nu^{'}},x_{\nu \nu^{"}}\right\}$, $\left\{{\bar{x}}_{\nu \nu^{'}},{\bar{x}}_{\nu \nu^{\prime \prime }}\right\}$ to *F*. This ensures that every vertex *v *in *G *is mapped to one and only one vertex in *H *(i.e., the map is a well-defined function). (We assume that |L(*v*)| ≠ 0; otherwise, the instance can be rejected.)

For every two vertices *v *and *u *in *G *such that there is a directed path from *v *to *u *in *G *(i.e., *v *appears before *u *in the directed path in *G*), and for very $\nu^{'}\in L\left(\nu \right)$ and $u^{'}\in L\left(u\right)$ such that $\nu^{'}=u^{'}$ or $u^{'}$appears before *u *in the directed path in *H*, we add the clause $\left\{{\bar{x}}_{\nu \nu^{'}},{\bar{x}}_{uu^{'}}\right\}$ to *F*. This ensures that the desired mapping is injective, and ensures that the mapping respects the precedence order among the vertices in *G *that is defined by the directed path in *G *(property (ii)).

For every two vertices *v *and *u *in G such that *vu *∈ *E*(*G*), and for very $\nu^{'}\in L\left(\nu \right)$ and $u^{'}\in L\left(u\right)$ such that $\nu^{'}u^{'}\notin E\left(H\right)$, we add the clause $\left\{{\bar{x}}_{\nu \nu^{'}},{\bar{x}}_{uu^{'}}\right\}$ to *F*. This ensures that the desired mapping respects the undirected edges of *G *(property (iii)).

This completes the construction of *F*. Clearly, this construction can be carried out in polynomial time.

It is not difficult to verify that (*G*, *H*) is a yes-instance of 2-GRAPH EMBEDDING if and only if *F *is a yes-instance of 2-CNF-SATISFIABILITY. This implies that 2-GRAPH EMBEDDING is polynomial-time solvable.   □

The above theorem, together with the result in [3], provides a complete characterization of the complexity (NP-hardness) of *r*-GRAPH EMBEDDING with respect to *r*.

If we consider the *r*-GRAPH EMBEDDING parameterized by *r*, the fact that the problem is NP-complete for *r *≥ 3 [3] implies that the problem is not solvable in time *O*(*n^r^*) unless P=NP, and hence, with respect to the parameterized complexity framework, the problem is not in the class XP. Therefore, there is not much hope behind seeking parameterized algorithms (with respect to *r*) for the problem. Moreover, the NP-hardness proof for *r*-GRAPH EMBEDDING (*r *≥ 3) is via a reduction from 3-CNF-SATISFIABILITY (each clause contains at most three literals) that produces two graphs *G *and *H*, each of size linear in the number of clauses of the 3-CNF-SATISFIABILITY instance. Therefore, based on the results in [23], we can conclude that *r*-GRAPH EMBEDDING (*r *≥ 3) is not solvable in subexponential time unless the exponential-time hypothesis (ETH) fails [23].

We investigate next the complexity of the *r*- GRAPH EMBEDDING_≥ _problem.

**Theorem 0.2 ***The r*-GRAPH EMBEDDING_≥ _*problem is *NP-complete, *for any r *≥ 1.

PROOF. It suffices to prove the NP-completeness of the 1-GRAPH EMBEDDING_≥ _problem. We only prove the NP-hardness, as it is very easy to show the membership of the problem in NP. The proof is via a reduction from the CLIQUE problem: Given a graph and a nonnegative integer k, determine if the graph has a clique (complete subgraph) of size *k*.

Let $\left(G^{'},k\right)$ be an instance of CLIQUE, where $V\left(G^{'}\right)=\left\{\nu_{1}^{'},...,\nu_{n}^{'}\right\}$. We construct the instance (*G*, *H*, *k*) of 1- GRAPH EMBEDDING_≥ _as follows. The set of vertices *V *(*G*) = {*v*_1_, ... ,*v_n_*} and *V *(*H*) = {*u*_1_, ... ,*u_n_*} are copies of $V\left(G^{'}\right)$. We connect the vertices *v*_1 _,..., *v_n _*in *G *by a directed path, and *u*_1_, ... ,*u_n _*in *H *by a directed path, and define $L\left(\nu_{i}\right)=\left\{u_{i}\right\}$, for *i *= 1, ... ,*n*. Finally, the undirected edges of *G *form a clique, and the undirected edges of *H *are those of $G^{'}$; that is, *v*_i_*v_j _*∈ *E*(*G*) for every 1 ≤ *i *≠ *j *≤ n, and *u_i_u_j _*∈ *E*(*H*) if and only if $\nu_{i}^{'}\nu_{j}^{'}\in E\left(G^{'}\right)$. This completes the reduction, which is obviously computable in polynomial time.

It is not difficult to verify that $\left(G^{'},k\right)$ is a yes-instance of CLIQUE if and only if (*G*, *H*, *k*) is a yes-instance of 1- GRAPH EMBEDDING_≥_. This completes the proof.   □

The reduction in the above theorem is an fpt-reduction, from the CLIQUE problem to 1- GRAPH EMBEDDING_≥_, where the parameter is the size of the subgraph sought *k*. Since CLIQUE is known to be *W *[1]-hard in the parameterized complexity hierarchy, we obtain:

**Theorem 0.3 **The *r*- GRAPH EMBEDDING_≥ _*problem is W *[1]-*complete*, *for any r *≥ 1. *(Note that membership in W *[1]*follows from the results in the next section.*)

Finally, we observe that the same reduction in Theorem 0.2 provides an *L*-reduction [24] (i.e., approximation-preserving reduction) from MAXIMUM CLIQUE (the problem of computing a clique of maximum cardinality in a graph) to MAXIMUM 1-GRAPH EMBEDDING. It is well known that, unless P=NP, MAXIMUM CLIQUE cannot be approximated to a ratio $n^{\frac{1}{2}-\varepsilon }$ for any *ε *> 0 [25]. It follows that:

**Theorem 0.4 ***Unless *P=NP, *the *MAXIMUM *r*-GRAPH EMBEDDING *problem cannot be approximated to a ratio *$n^{\frac{1}{2}-\varepsilon }$*for any ε *> 0.

### Graph embedding to independent set

In this section we show that the MAXIMUM *r*-GRAPH EMBEDDING problem can be modeled as an MAXIMUM INDEPENDENT SET problem. Recall that an *independent set *in a graph is set of vertices such that no two of them are adjacent, and the MAXIMUM INDEPENDENT SET problem asks for an independent set of maximum cardinality in a graph.

Let (*G*, *H*) be an instance of MAXIMUM *r*-GRAPH EMBEDDING. Suppose that *V *(*G*) = {*g*_1_, *g*_2_, ... ,*g_n_*} with directed edges from *g_i _*to *g_i_*_+1_, for 1 ≤ *i *≤ *n *− 1, and suppose that *V *(*H*) = {*h*_1_, *h*_2_, ... ,*h_m_*}, *m *≥ *n*, and with directed edges from *h_i _*to *h_i_*_+1_, for 1 ≤ *i *≤ *m *− 1. Suppose that each vertex of *G *can be mapped to one of at most *r *vertices in *H*.

**Theorem 0.5 ***If *MAXIMUM INDEPENDENT SET *is solvable in time *2*^cn^*, *then *MAXIMUM *r*-GRAPH EMBEDDING *is solvable in *2*^crn ^time*.

PROOF. Create an auxiliary graph *X *as follows. For each possible choice mapping *g_i _*to *h_j_*, create a vertex *x_ij_*. For any two vertices *x_ij _*and *x_kl_*, add an edge between them if and only if one of the following conditions are true:

1. *i *= *k *or *j *= *l*.

2. *i *<*k *and *j *>*l*, or *i *>*k *and *j *<*l*.

3. There is an undirected edge between *g_i _*and *g_k _*in *G*, while there is no undirected edge between *h_j _*and *h_l _*in *H*.

Note that Condition 2 could be removed when the order of the mapped vertices are not required to be the same for the two graphs.

It is clear that any independent set of *X *corresponds to a common subgraph of *G *and *H *of the same size. So the problem of finding a maximum common subgraph of *G *and *H *is reduced to the problem of finding a maximum independent set of *X*, which has *rn *vertices. In particular, to find if *G *is a subgraph of *H *it suffices to find an independent set of size *n*. Therefore if MAXIMUM INDEPENDENT SET is solvable in time 2*^cn^*, then MAXIMUM *r*-GRAPH EMBEDDING is solvable in 2*^crn ^*time.   □

If we use the current-best exact algorithm for MAXIMUM INDEPENDENT SET by Robson [26] that runs in time *O*(2*^n^*^/4^), we conclude that:

**Theorem 0.6 ***The *MAXIMUM *r*-GRAPH EMBEDDING *problem is solvable in time O*(2*^rn^*^/4^), *where n is the number of vertices in graph G*.

### Algorithm for structure comparison

The problem of protein structure comparison could be modeled as finding an independent set problem of an auxiliary graph. When aligning two protein structures, the auxiliary graph *X *is created as is in the proof of Theorem 2.5. Note that when aligning three and more protein structures, the auxiliary graph *X *could be created similarly.

Refer to the following for the outline of the algorithm for protein structure comparison.

1 *(Preprocessing)*. Generate the two structure graphs for the two proteins, based on both their secondary structure information (local structure) and tertiary structure (global structure) information.

2 *(Auxiliary graph)*. Build the auxiliary graph from the two structure graphs;

3 *(Top K independent sets)*. Generate the top *K *maximum independent sets of the auxiliary graph by applying the enumeration algorithm developed in [4].

4 *(Matched SSEs)*. Evaluate the generated top *K *maximum independent sets and generate the SSE pairs with the best score of the two proteins.

We analyze the time complexity of the algorithm:

Step 1: The algorithm processes the two proteins to generate the corresponding two structure graphs, where each vertex of a graph represents an SSE of the corresponding protein. Suppose the number of the vertices of each structure graph is bounded by *n*.

Step 2: We introduce a parameter *r *as the maximum number of pairs associated with each vertex of the structure graphs. The number of vertices of the auxiliary graph is bounded *rn*.

Step 3: Through calling the enumeration algorithm develop in [4], it takes time *O*(1.47*^rn^*) to generate the top *K *independent sets of the auxiliary graph.

Step 4: It takes time *O*(1.47*^rn^n*^2^) to evaluate the generated independent sets and identify the independent set, which corresponding to the SSE pairs with the best score of the two proteins.

Refer to [27] for a discussion of the theoretical running times of several other graph-based approaches for protein structure comparison, which are of *O*((*mn*)*^n^*) or *O*(*m^n^*^+1^)*n*), where *m *and *n *demote the size of the structure graphs. The theoretical running-time *O*(1.47*^rn^n*^2^) of our approach ePC is the current best, where *n *is the smaller number of SSEs of the two proteins, *r *is a parameter of small values.

## Testing results

Our approach ePC is designed for general-purpose protein structure comparison. In this section we test our approach for this purpose using SABmark-sup and SABmark-twi [28], and specific novel folds studied in the literature. Our approach is implemented using C++. The testing is mainly performed on a regular Macbook (8GB Mem). The running-time testing is conducted on a Dell server (PowerEdge 2950III, 32GB Mem). Due to the space limit, some testing results are not presented.

Given two proteins, *A *and *B*, the score of the a SSE pair is the sum of the *L_ij _*of the residues for the SSE pair. *L_ij _*is defined in [29] denoting the similarity between a segment centered around residue *i *of one protein and a segment centered around residue *i *of the other protein, where $L_{ij}=\text{min}\left\{D\left(d_{i-2,i+2}^{A},d_{j-2,j+2}^{B}\right),D\left(d_{i-2,i+1}^{A},d_{j-2,j+1}^{B}\right),D\left(d_{i-1,i+2}^{A},d_{j-1,i+2}^{B}\right)\right\}$, where *D*(*d*_1_, *d*_2_) = 0.1 − |*d*_1 _− *d*_2_|/(*d*_1 _+ *d*_2_).

Let *S *be the sum of the scores of all the aligned SSEs. The normalized score $S_{n}=S/\sqrt{\left(l_{A}*l_{B}\right)}$, where *l_A _*and *l_B _*are the lengths of the two proteins. *A_c _*is the number of SSEs in *A*, *B_c _*is the number of SSEs in *B *and *MCS_n _*is the size of the common subgraph of the two protein structure graphs, the CORE-COV is a percentage defined by: *MCS_n _*/ min(*A_c_*, *B_c_*).

### Testing different parameter values

There are two important parameters of our algorithm *r *and *K*, where *r *is the maximum number of SSE pairs associated with each SSE of the structure graphs, and *K *is the number of enumerated independent sets. Note that the score *L_ij _*of the SSE pairs is the criteria for identifying the associated *r *SSEs. We test the impacts of the two parameter values on the running time and scoring for the protein comparison.

**Figure 2:**
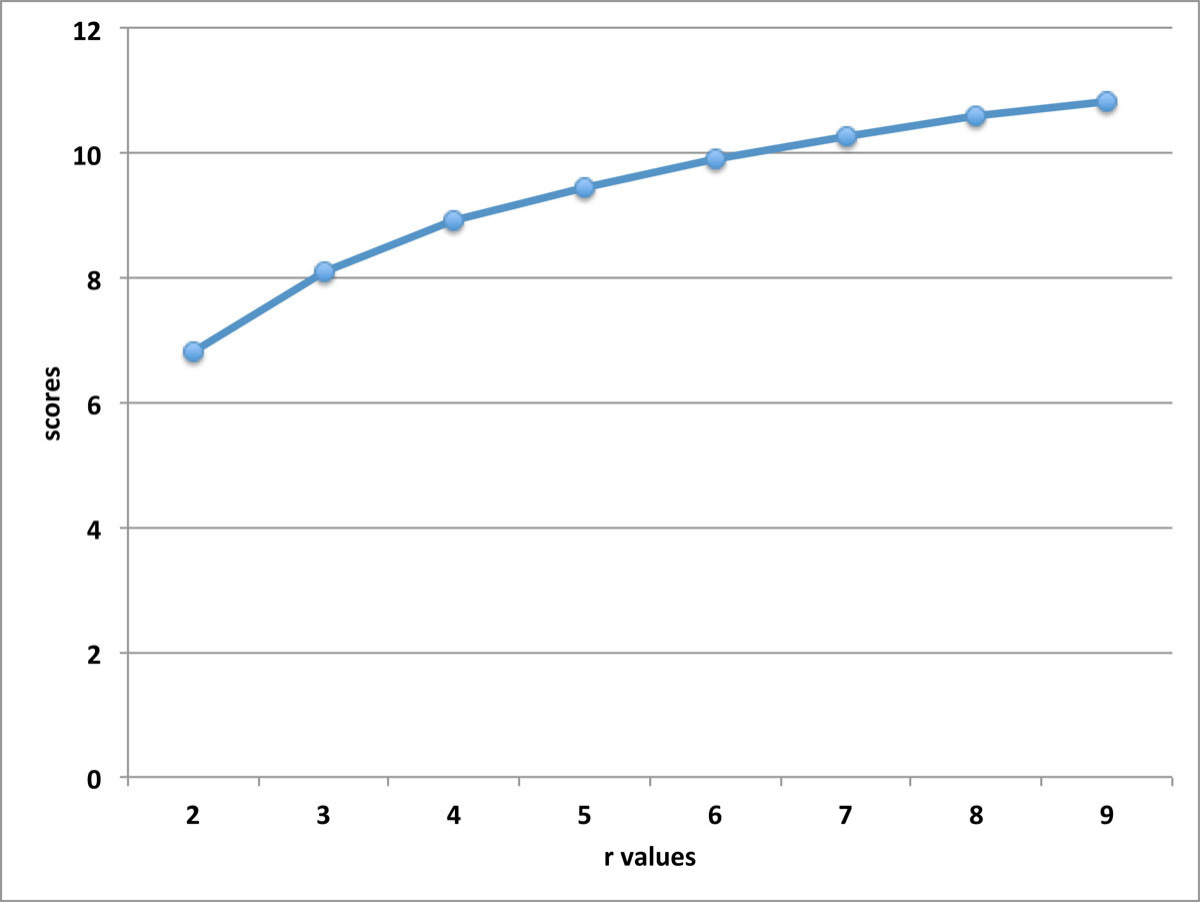
**The running times for different r values**. Note for all these testing, our approach use the same parameter K = 1000.

**Figure 3:**
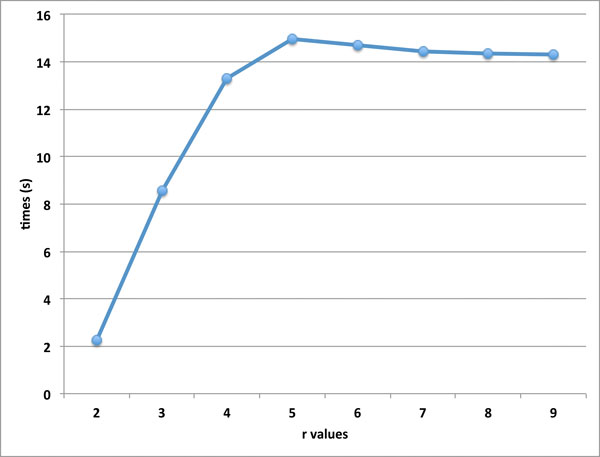
**The scores for different r values**. Note for all these testing, our approach use the same parameter K = 1000.

We present our testing results for accuracy (using the score *S *as a criteria) and running-time of our approach with different parameter *r *values. We have conducted the testing of 200 protein pairs from SABmark-sup database with different parameter *r *values, where each SSE from one protein is matched with the top *r *SSEs from the other protein. Refer to Figure 2 for the average scores of 200 protein pairs from Sup database, when testing our approach with different parameter *r *values, *r *= 2, 3, 4, 5, 6, 7, 8, 9. Our testing results indicate that when the parameter *r *value increases, the score has increased. Refer to Figure 3 for the average running times of 200 protein pairs from Sup database, when testing our approach with different parameter *r *values. When *r *increases, the running time of our approach increases in general. However note that the running times when *r *= 5, 6, 7, 8, 9 are very similar; this is because trimming has been applied to reduce the sizes of the auxiliary graphes before the enumeration of the independent sets, and also because the impact of the parameter *K *on the running time. Especially the running time when *r *= 2 is significantly lower than the other cases, which matches our theoretical result that for *r *= 2 the *r*-GRAPH EMBEDDING problem is in P.

For the enumeration of independent sets, we have introduced a parameter *K*, which is the bound of the number of enumerated independent sets. Here we present our testing results for accuracy and running-time of our approach with different parameter K values (See Table 1). Similar as the testing for the parameter *r*, we have conducted the testing of 200 protein pairs from SABmark-sup database with different parameter *K *values, *K *= 125, 250, 500, 1000. Our testing results indicate that when the parameter *K *value increases, the score has increased and the running time also increases.

**Table 1:** The running times and scores for different *K *values

K =	125	250	500	1000
time	1.90	3.57	6.76	12.73
score	8.89	9.08	9.17	9.28

Note for all these testing our approach use the same parameter *r *= 6.

### Performing structure comparison

*Self-querying in a large database of structures*. As pointed in [5], a necessary condition for a approach to be of practical value for structure comparison and classification, it should be able to find the query itself in a database of protein structures. To test this property of our approach, 1000 protein structures from the SABmark-sup database. Our approach with the normalized score function can identify the query structure with ranking No. 1 with 100% accuracy.

*Detecting a substructure in a set of larger structures*. Our approach can detect a smaller query structure (or, a motif structure) within a larger target structure. We use the test set from the previous test and required for each domain to be matched to the target domain embedded in the original full-protein structure. Our approach with the normalized score can identify the substructure with ranking No. 1 with 100% accuracy.

*Protein family classification*. We compare the performance of our approach for protein family classification with deconSTRUCT, which is also an SSE-based method and designed for protein structure database filtering. We have tested 1000 proteins pairs of the SABmark [28]. Due to the space limit, we only discuss some of the representative testing result. We align protein d1a6m (core size:7; AAs: 151; from SABmark [28]) to proteins from 10 different families of the twi database with each family 10 proteins. Of the proteins in the top 10 ranking, 7 proteins identified through our approach are the proteins from the same family as protein d1a6m. For deconSTRUCT, 7 proteins of the identified 10 proteins (without ranking) are the proteins from the same family as protein d1a6m. Form the testing results, our approach has comparable performance with DeconSTRUCT for the general purpose protein structure comparison and structure classification. The mixed graph representation of our approach ePC is much simpler compared with deconSTRUCT. Our approach ePC is more flexible than deconSTRUCT in that ePC can handle SSE alignments with and without respect to the order of SSEs, which will be discussed in the next section for specific examples.

### Specific examples

We test our approach on specific examples for common substructures and novel folds which share common substructures with non-sequential SSEs.

*Detection of several different common substructures*. We test our approach ePC using the four protein structures (PDB codes: 1a02N, 1iknA, 1nfiA, and 1a3qA) studied in [8,9]. The proteins share two common domains: "p53-like transcription factors" and "E set domains". In [8,9] two different common substructures were detected, one for each domain. The first common substructure is part of the "p53-like transcription factors" domain. It consists of 114 residues, and it forms a sandwich of nine *beta*-strands. The second common substructure is part of the "E set domains" domain. It consists of 87 residues, and it forms a sandwich of seven *beta*-strands.

**Figure 4:**
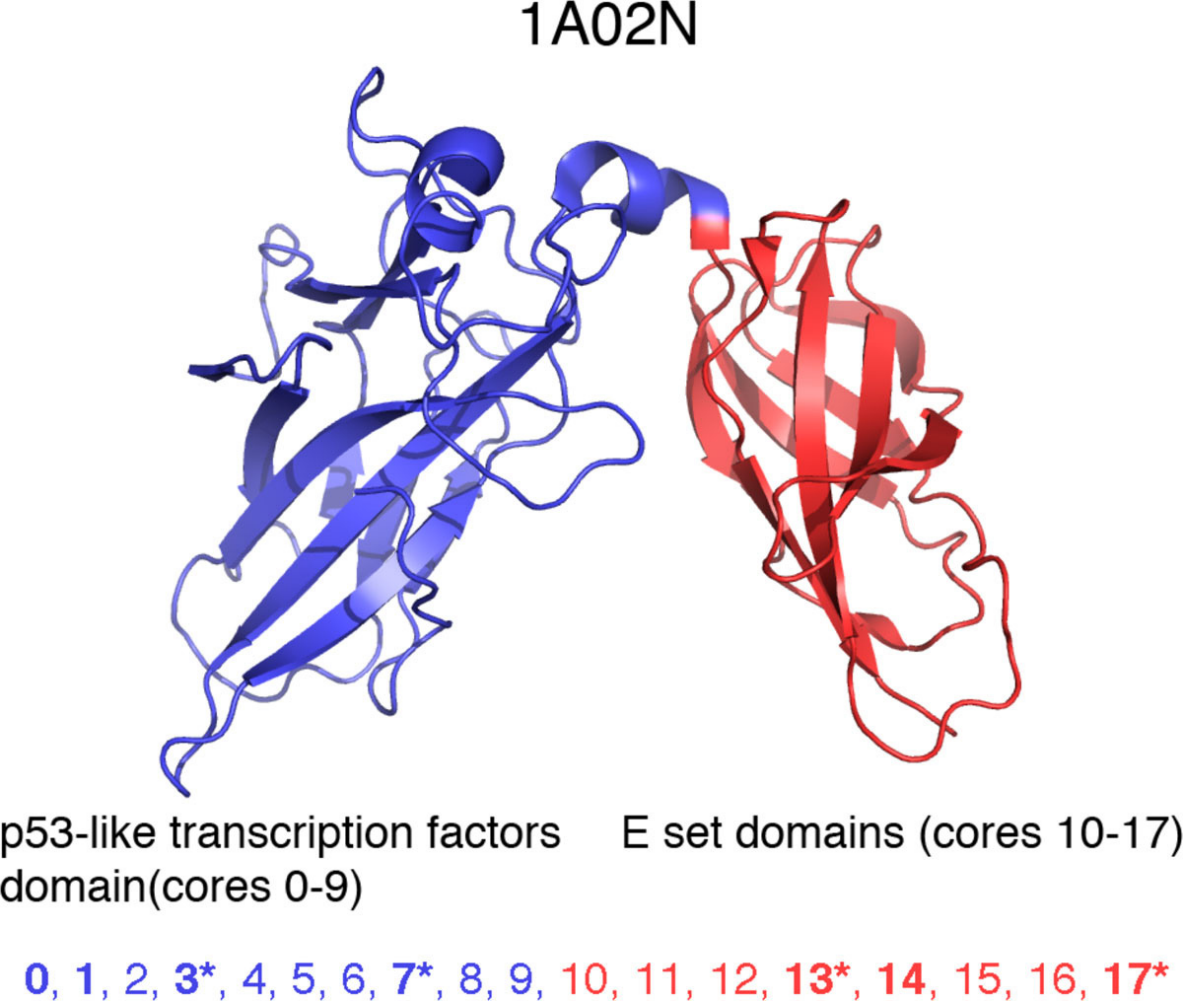
**The 3D Structure of 1a02N with its two domains: p53-like transcription factors and E set domains**. There are 18 cores/SSEs (0-17) with conserved SSEs marked with *. Matched SSEs of 1a02N and 1ikna: (0,1) (1,2) (3,3) (7,5) (13,7) (14,8) (17,11); Matched SSEs of 1a02N and 1nfia: (0,1) (1,2) (3,3) (7,5) (12,7) (13,10) (15,11) (17,13); Matched SSEs of 1a02N and 1a3qa: (3,0) (5,3) (6,4) (7,5) (13,7) (14,8) (16,12) (17,13).

Please refer to the following testing results of our approaches, when 1a02N is compared with: 1iknA, 1nfiA, and 1a3qA. Our testing results match the results in [8,9]. Especially for the second common substructure that is part of the "E set domains" domain with conserved matched SSEs: 12, 13, 14, 15, 16, 17 of 1a02N. Please refer to Figure 4 for its 3D structure and the two domains.

**Figure 5:**
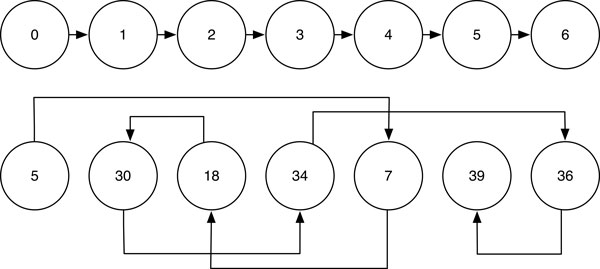
**Structure alignment of PDB:2AJE and PDB:1J7NB**. Structure alignment of the new fold PDB:2AJE and the structural analog PDB:1J7NB, showing nonsequential order of aligned SSEs.

**Figure 6:**
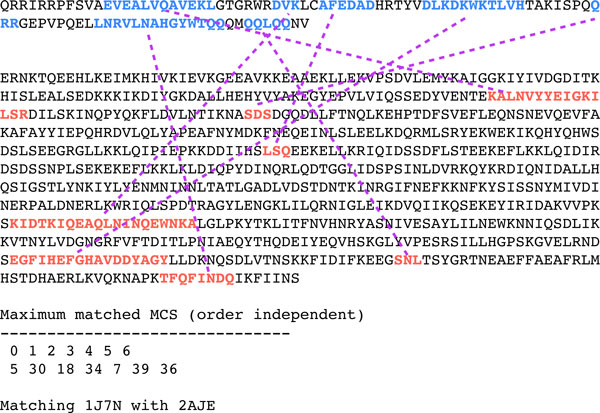
**Aligned SSEs of PDB:2AJE and PDB:1J7NB**. The amino acid sequences of the new fold PDB:2AJE and the structural analog PDB:1J7NB, showing the non-sequential order of aligned SSEs of the two protein sequences.

*Three novel folds*. The three novel folds were discussed in [7] to study the unique feature of GANGSTA+ to conduct non-sequential SSE alignment. Note that the protein structures that are structurally similar to the listed three new folds were detected through scanning the ASTRAL40 database by GANGSTA+. The detected similar protein structures have non-sequential SSE alignments with the three novel folds respectively. Please refer to our testing result in Table 2 Figure 5 and 6.

**Table 2:** Structure search and comparison of the three novel folds with the structural analogs

New fold	Detected analog	DaliLite	TM-align	GANGSTA+	deconSTRUCT	SSM	ePC
2JMK/7/57	1GO4H/4/93	11.0/0/75	4.0/1/67	1.8/7/61	0/0	1/14	4/100%/8.3
2AJE/7/44	1J7NB/40/738	3.9/3/45	3.4/3/45	2.1/4/53	3/31	3/61	7/100%/10.9
2ES9/5/58	1SXJH/15/267	2.5/4/57	4.0/5/65	1.8/5/69	3/36	2/38	5/100%/9.9

The results for DaliLite, TM-align and GANGSTA+ are from [7]. The format of protein entries: PDB ID/number of SSEs/number of residues in these SSEs. The format of the testing result for DaliLite, TM-align and GANGSTA+: RMSD/number of aligned SSEs/number of aligned residues. The format of the testing results for deconSTRUCT and SSM: no. of matched SSEs/no. of aligned AAs, and for our approach ePC: no. of matched SSEs/CORE-COV/score).

## Discussion

We use an SSE-based graph model for general purpose protein structure comparison. We presented the computational complexity results related to the protein structure comparison problem. An effective algorithm is developed integrating a novel enumeration of independent sets and parameterized computation for the problem. Our approach is tested for protein structure comparison using benchmark testing sets. Compared with other SSE-based approaches, our approach has comparable performance for the general purpose protein structure comparison. We also demonstrate that our approach could be applied to identify common substructure with non-sequential SSEs and proteins sharing more than one common substructure.

## Competing interests

The authors declare that they have no competing interests.

## Authors' contributions

XH, IK and GX carried out the study on the complexity and the design of the approach for the protein structure comparison problem, and drafted the manuscript. CA, DJ and KW participated in the implementation and the testing of the algorithm. All authors have approved the final manuscript.

## Declarations

The publication costs for this article were funded by the corresponding author's institution.

This article has been published as part of *BMC Genomics *Volume 14 Supplement 2, 2013: Selected articles from ISCB-Asia 2012. The full contents of the supplement are available online at http://www.biomedcentral.com/bmcgenomics/supplements/14/S2.
